# PCDD and PCDF exposures among fishing community through intake of fish and shellfish from the Straits of Malacca

**DOI:** 10.1186/s12889-015-2044-3

**Published:** 2015-07-21

**Authors:** Azrina Azlan, Nurul Nadiah Mohamad Nasir, Norashikin Shamsudin, Hejar Abdul Rahman, Hock Eng Khoo, Muhammad Rizal Razman

**Affiliations:** Department of Nutrition and Dietetics, Faculty of Medicine and Health Sciences, Universiti Putra Malaysia, 43400 UPM Serdang, Selangor Malaysia; Research Centre of Excellence for Nutrition and Non-communicable Disease, Faculty of Medicine and Health Sciences, Universiti Putra Malaysia, 43400 UPM Serdang, Selangor Malaysia; Department of Medicine, Faculty of Medicine and Health Sciences, Universiti Putra Malaysia, UPM Serdang, 43400 Selangor Malaysia; Department of Community Health, Faculty of Medicine and Health Sciences, Universiti Putra Malaysia, UPM Serdang, 43400 Selangor Malaysia; Research Centre for Sustainability Science and Governance (SGK), Institute for Environment and Development (LESTARI), Universiti Kebangsaan Malaysia, UKM Bangi, 43600 Selangor Malaysia

**Keywords:** PCDD, PCDF, Congener, Fish, Shellfish, Fisherman, Skin disease

## Abstract

**Background:**

Exposure to PCDD/PCDF (dioxin and furan) through consumption of fish and shellfish is closely related to the occurrence of skin diseases, such as chloracne and hyperpigmentation. This study aimed to determine the exposure of PCDD/PCDF and its congeners in fish and shellfish obtained from different regions of the Straits of Malacca among the fishing community.

**Methods:**

The risk of fish and shellfish consumption and exposure to PCDD/PCDF among fishermen living in coastal areas of the Straits were evaluated based on a cross-sectional study involving face to face interviews, blood pressure and anthropometric measurements, and administration of food frequency questionnaires (FFQ). Skin examination was done by a dermatologist after the interview session. Determination of 17 congeners of PCDD/PCDF in 48 composite samples of fish and shellfish was performed based on HRGC/HRMS analysis.

**Results:**

The total PCDD/PCDF in the seafood samples ranged from 0.12 to 1.24 pg WHO-TEQ/g fresh weight (4.6-21.8 pg WHO-TEQ/g fat). No significant difference found for the concentrations of PCDD/PCDF between the same types of seafood samples obtained from the three different regions. The concentrations of the most potent congener, 2,3,7,8-TCDD in the seafood samples ranged from 0.01 to 0.11 pg WHO-TEQ/g FW (1.9 pg WHO-TEQ/g fat). A positive moderate correlation was found between the fat contents and concentrations of PCDD/PCDF determined in the seafood samples. The total PCDD/PCDF in all seafood samples were below the 1 pg WHO-TEQ/g fresh weight, with the exception of grey eel-catfish. The respondents had consumed fish and shellfish with the amounts ranging between 2.02 g and 44.06 g per person per day. The total PCDD/PCDF exposures through consumption of fish and shellfish among the respondents were between 0.01 and 0.16 pg WHO-TEQ/kg BW/day. With regard to the two PCDD/PCDF-related skin diseases, no chloracne case was found among the respondents, but 2.2 % of the respondents were diagnosed to have hyperpigmentation.

**Conclusion:**

Intake of a moderate amount of fish and shellfish from the area is safe and does not pose a risk for skin diseases. An over-consumption of seafood from the potentially polluted area of the Straits should be monitored in future.

## Background

Fish and shellfish are the richest sources of long-chain (LC) *n*-3 polyunsaturated fatty acids (PUFA), such as eicosapentaenoic acid (EPA) and docosahexaenoic acid (DHA) [1]. In Malaysia, fish consumption among Malaysians ranked the second (40.78 %) of the top ten daily consumed foods [2]. Although marine fish contains a high level of PUFA, the persistent chemical contaminants are the main problem for fish consumption [3]. Industrialisation has polluted seawater with chlorinated organic compounds and precursors of polychlorinated dibenzo-p-dioxins (PCDD) or polychlorinated dibenzofurans (PCDF). These compounds are persistent organic pollutants (POP) that have a high tendency to be accumulated in the tissues of fish and shellfish [4].

It is well known that marine sources (fish and shellfish) or marine products constitute an important route of human exposure to PCDD/PCDF and other persistent organic chemicals [5–7]. Fish and shellfish may bioaccumulate POP in their tissues. Eating this seafood potentially transfers POP to the human body [8–11]. There is a distinct pattern of accumulation of POP in the human body, which depends on factors such as types of fish and shellfish consumed [12].

Severe exposure to PCDD/PCDF (dioxin and furan) would pose adverse health effects to human, such as chloracne (a skin disease), discolouration of the skin, rashes, liver damage, reproductive and developmental effects, and cancer [13]. Chloracne, hyperpigmentation and hirsutism are the most widely recognised skin diseases, and consistently observed features due to high exposure to 2,3,7,8-tetrachlorodibenzo-*p*-dioxin (TCDD) [14, 15]. Chloracne is often accompanied by a detectable high level of PCDD/PCDF in human blood that orally ingested compared with dermal contact [14].

The recommended level of the tolerable daily intake (TDI) for PCDD/PCDF in food is <1 pg TEQ/kg body weight (BW) [16]. In fish, the level of PCDD/PCDF at about 1 pg WHO-TEQ/g fat is considered safe [17]. WHO experts suggested the toxic equivalency factor (TEF) values (≤1) for PCDD/PCDF in food. These factors are used to calculate WHO toxicity equivalents (WHO-TEQ) for mixtures of PCDD/PCDF. WHO-TEQ can then be used to express the estimated combined toxicity of the mixture relative to the lead component 2,3,7,8-TCCDD. WHO-TEQ has been used for estimating toxicity risk for PCDDs and PCDFs. Moreover, the recommended TEF levels for the 17 congeners of PCDD/PCDF in food are 0.0001-1.0 [18]. The Malaysian Food Act (1983) has underlined the maximum level of certain chemicals detected in food for human consumption. The chemicals covered under the Act are antibiotic residues, drug residues, food additives and pesticide residues [19]. However, no chemical contaminants have been specified. Dietary exposure to contaminants including PCDD/PCDF is also not stated in any of the dietary guidelines.

Due to the toxic effect of PCDD and PCDF found in most of the seafood obtained from marine sources, the study was aimed to determine the types (congeners) and quantities of PCDD/PCDF in marine fish and shellfish. Investigation of the occurrence of skin diseases among fishing communities along the Straits of Malacca was also performed in relation to dietary PCDD/PCDF exposure. As efforts intensify, the results obtained from this study can be used as a reference for monitoring seafood quality at the local markets.

## Methods

### Chemicals and standards

Solvents (dichloromethane-DCM, toluene and hexane), which were of pesticide grade, were purchased from the Fisher Scientific (Leicestershire, UK) and hydromatrix was obtained from Frampton Ave (Harbor City, CA, USA). Calibration standard EDF-9999, ^13^C_12_-labelled internal standard EDF-8999 and recovery standard EDF-5999 were supplied by Cambridge Isotope Laboratories, Inc. (Andover, MA, USA). All other chemicals used were of analytical grade.

### Sample collection and preparation

**Fig. 1:**
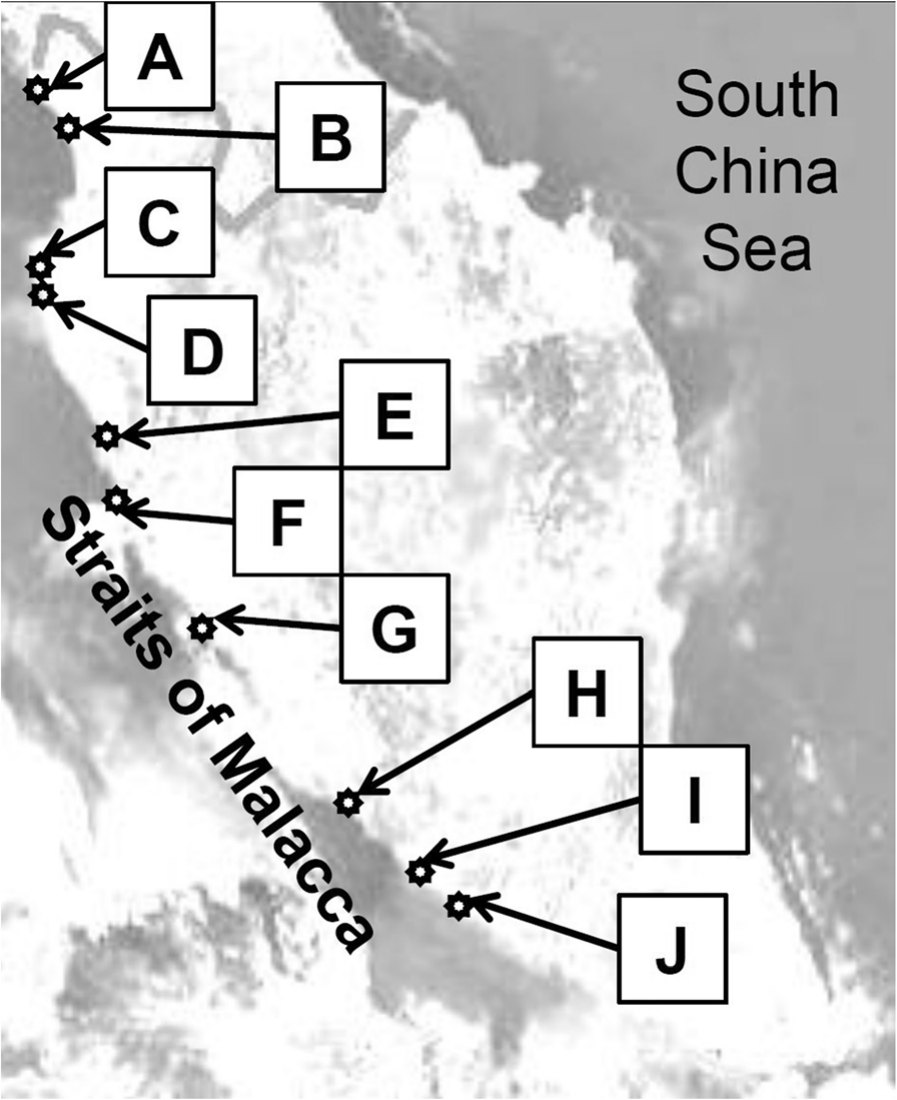
Locations of fish landing areas along the Straits of Malacca

Fresh samples consisted of 48 samples from fifteen different types (species) of fish (12 types) and shellfish (three types). The samples were collected from three different regions of the identified fish landing areas along the Straits of Malacca. As shown in Fig.  1, the three regions were the northern region [Kuala Perlis – A (6° 24′ 02.0″ North, 100° 07′ 49.4″ East), Kuala Kedah – B (6° 06′ 23.7″ North, 100° 17′ 18.1″ East), Teluk Bahang – C (5° 27′ 35.3″ North, 100° 12′ 39.6″ East) and Pulau Betong – D (5° 18′ 25.3″ North, 100° 11′ 36.4″ East)], middle region [Pengkalan Baharu – E (4° 26′ 41.7″ North, 100° 36′ 59.5″ East), Kuala Sepetang – G (4° 50′ 05.9″ North, 100° 37′ 38.2″ East), and Kuala Selangor – F (3° 21′ 10.8″ North, 101° 14′ 53.9″ East)], and southern region [Melaka – H (2° 10′ 58.6″ North, 102° 15′ 58.6″ East), Port Dickson – I (2° 31′ 18.5″ North, 101° 47′ 46.7″ East) and Muar – J (2° 02′ 55.5″ North, 102° 33′ 09.6″ East)]. All fish samples were collected in August 2008 (trip 1) and November 2008 (trip 2), and duplicate samples were obtained from each trip [20]. The samples were collected during two different trips (trip 1 = T1; trip 2 = T2), due to availability of samples for each type of the seafood at the time of collection, with the help of officers from the Fisheries Development Authority of Malaysia (FDAM). No permission was required for the seafood sample collection at any of the fish landing areas.

The marine fish and shellfish samples consisted of the following species: Indian mackerel, Spanish mackerel, silver pomfret, hardtail scad, fourfinger threadfin, dorab wolf-herring, large-scale tongue sole, long-tailed butterfly ray, Japanese threadfin bream, sixbar grouper, Malabar red snapper, grey eel-catfish, cockles, prawn and cuttlefish. The collected seafood species were the commonly consumed species and did not involve any protected or endangered species. The duplicate samples were obtained from the same type of fish or shellfish in the same region. Each composite sample contained 10 g of fresh fish fillet or muscle tissue of shellfish. Duplicate composite samples of the fish or shellfish samples were obtained during Trip 1 (T1) and Trip 2 (T2).

The fish and shellfish samples collected were transported to nutrition laboratory on the same day of collection. The samples were delivered to the laboratory in sealed polystyrene boxes and stored in a freezer (–20 °C). Before the analysis, the whole fish was weighed, gutted, washed and filleted. The edible parts of prawn and cockle were obtained and washed before analysis. The prepared samples were stored in polyester covered cups at –20 °C before further analyses. Samples were also sent to the Doping Control Centre, Penang (an accredited laboratory) for determination of PCDD and PCDF congeners.

### Fat extraction

Before the extraction, the frozen samples were thawed at room temperature and 50 μl of C_13_-labelled internal standard (EDF-8999) was spiked into the 10 g sample. The sample was then mixed with 10 g of hydromatrix before homogenisation using a mortar. The homogenised sample then was dried in an oven at 50 °C for 2 min to remove the moisture. The dried sample was powdered, placed into a cell (size 33) and covered with Ottawa sand (Fisher Scientific, Leicestershire, UK) before extraction using an Accelerated Solvent Extraction System (ASE 200) (DIONEX Corporation U.S Patents, Sunnyvale, USA) for 20 min based on USEPA Method 3545 [21]. The fat was extracted using DCM, and the DCM was removed using a rotary evaporator (BUCHI Labortechnik, Flawil, Switzerland) for 20 min at 40 °C. The remaining was filtered to obtain a crude fat extract. The fat content was determined gravimetrically.

### Clean-up process

Hexane was added to the crude fat extract, forming an aliquot. The aliquot was placed in a fully automated Power-Prep Fluid Management System (FMS) (Fluid Management System, Inc., Waltham, USA) for extract clean-up. The process involved three types of columns, silica (CLDS-ABN-STD), alumina (CLDA-BAS-011) and carbon (CLDC-CCE-034). The column chromatographic clean-up procedure was adapted from the Smith-Stalling method outlined by the US EPA Method 8290. After completion of FMS clean-up, the eluent that contained PCDD/PCDF was concentrated to approximately 1 ml, and later spiked with 50 μl of the external standard (EDF-5999). The spiked eluent was then micro-concentrated by using a Dri-Block Heater DB-20 (Staffordshire, OSA, UK) and the nitrogen gas from TESCOM Corporation (ELK River, MN, USA) at 60 °C to a final volume of 10 μl. The eluent was placed in a 1.5-ml aluminum-covered vial before HRGC/HRMS analysis.

### Determination of PCDD/PCDF

HRGC/HRMS system from Thermo Scientific (Milan, Italy) was applied in this study. The HRGC/HRMS analysis was coupled with mass spectrometry (MS) from Thermo Fisher Scientific (Bremen, Germany), and it was used for determination of PCDD and PCDF. Each sample was determined for its 17 PCDD/PCDF congeners with 2,3,7,8-chloro-substitution (seven PCDD and ten PCDF congeners). Concentrations in fish and shellfish samples were calculated based on fresh weight (FW) basis. The WHO toxicity equivalent (WHO-TEQ) for PCDD/PCDF in the seafood samples was calculated by applying the WHO 2005 toxic equivalency factor (TEF) for PCDDs and PCDFs [18]. This standard set of WHO-TEQ was used to evaluate the toxic effect of the seventeen most toxic PCDD/PCDF congeners found in the fish and shellfish samples. WHO-TEQ of the samples was calculated by multiplying the absolute concentration of each congener by a numeric factor that expresses the concentration in terms of the dioxin molecule, 2,3,7,8-TCDD, which is given a value of 1. Any values of PCDD/PCDF congeners below the limit of detection (LOD) should be reported as not detected.

### Dietary exposure to PCDD/PCDF

A randomised cross-sectional study was designed to assess the dietary exposure to PCDD/PCDF of fishermen living in the seaside of the Straits of Malacca. The respondents (n = 93) of this study were randomly selected from an identified fishing community in Kuala Selangor, Selangor, Malaysia and the survey area was permitted by the FDAM. The survey was performed at the office of Kuala Selangor Fishermen’s Association. The respondents were selected based on the inclusion criteria. Only healthy fishermen aged 18-55 years old without chronic diseases were selected. They were requested to fill a consent form, as well as subject information sheet, before undergoing the interview process. Ethical approval was obtained from the Human Medical Research Ethics Committee of Universiti Putra Malaysia [UPM/FPSK/PADS/T7-MJKEtikaPer/F01-JPD_JAN(10)03].

In this study, a Malay language version of the questionnaire was used. The survey was initiated after written informed consent was obtained from all respondents. Trained interviewers conducted the interviews, performed blood pressure and anthropometric measurements (body fat percentage, weight and height), and administered food frequency questionnaires (FFQ). The frequencies of consumption of the specific seafood were estimated based on the FFQ consumed on a daily, weekly or monthly basis. Skin physical examination was done by a dermatologist after the interview. The average seafood consumption (ASC) of fish and other types of seafood was calculated based on the consumption of fish and shellfish per day, and the level of PCDD/PCDF exposure from the intake of fish and shellfish was determined using the formula [7] as follows:

$$ \mathrm{PCDD}/\mathrm{PCDF}\ \mathrm{exposure}=\frac{\left[\mathrm{A}\mathrm{S}\mathrm{C}\ \left(\mathrm{g}/\mathrm{day}\right)\right] \times \left[\mathrm{PCDD}/\mathrm{PCDF}\ \mathrm{level}\ \mathrm{in}\ \mathrm{fish}\ \mathrm{and}\ \mathrm{shellfish}\ \left(\mathrm{pg}/\mathrm{g}\right)\right]}{\mathrm{Body}\ \mathrm{weight}\ \mathrm{of}\ \mathrm{each}\ \mathrm{respondent}\ \left(\mathrm{kg}\right)} $$

### Quality control

Method validation was done for the analysis of PCDD/PCDF in the samples. A calibration standard was used to construct a calibration curve based on the EDF-4141 window-defining standard in Xcalibur software. Reproducible calibration and testing of the extraction, cleanup, and HRGC/HRMS system were done to ensure the quality of the analysis. Sensitivity, linearity and repeatability of the instrument performance were also checked using the calibration standard. The acceptable recovery was within the range of 55-120 %. For dietary exposure, the newly designed questionnaire was pretested using a convenient sampling through an interview session among fishermen (n = 23) at the first visit of study location prior the actual data collection. The pilot test was to evaluate the acceptance of the questionnaire in terms of language, meaning, uses of words and other aspects. All questionnaires were checked for readability and missing data prior to data entry.

### Data analysis

The data obtained from the survey were analysed using the Statistical Package for Social Science (SPSS) programme version 20.0. All the data were presented as mean ± standard error of the mean (SEM). The ranges of values for fat content, PCDD/PCDF level, fish and shellfish intake, and PCDD/PCDF exposure were obtained. The survey data (baseline characteristics, fish and shellfish consumption, and skin diseases) were presented as percentage, mean ± SEM and range. Statistical analysis of the fish and shellfish samples was performed by applying independent sample *t*-test (comparing between different regions). Pearson’s correlation analysis was performed for the fat content and PCDD/PCDF level analysed. The concentrations of PCDD/PCDF in fish and shellfish samples were reported as pg WHO-TEQ/g FW. LOD was used for value below the detection limit (0.001 pg/g) and was presented as not detected.

## Results and discussion

### Levels of PCDD/PCDF and congeners profile

Tables 1, 2, 3 show the concentrations of PCDD/PCDF congeners in the fish and shellfish samples. The concentrations of PCDD/PCDF congeners were presented as levels of WHO-TEQ (pg/g) that contributed to the PCDD/PCDF toxicity. The concentrations of PCDD/PCDF in the samples between T1 and T2 were almost similar, except for Japanese threadfin bream (T1 = 0.18 pg/g; T2 = 0.73 pg/g) and grey eel-catfish (T1 = 0.90 pg/g; T2 = 1.57 pg/g). The exception may indicate the sporadic occurrences of these contaminants that may be due to unpredictable pollutants or spoilage events along the Straits of Malacca.

**Table 1:** Congeners of PCDD/PCDF in fish and shellfish samples from trip 1 (T1) and trip 2 (T2) of northern region

Congeners	SP*		HS*		FT*		IM*		LTS*		LBR*		Cf*		JTB*		SG*	
	T1	T2	T1	T2	T1	T2	T1	T2	T1	T2	T1	T2	T1	T2	T1	T2	T1	T2
2,3,7,8-TCDF*	ND*	0.01	ND	ND	ND	ND	0.01	ND	ND	ND	ND	ND	ND	ND	0.01	0.61	ND	ND
2,3,7,8-TCDD*	0.02	0.02	0.02	0.02	0.01	0.02	0.02	0.02	0.02	0.02	0.01	0.02	0.01	0.02	0.04	0.02	0.01	0.02
1,2,3,7,8-PeCDF*	ND	ND	ND	ND	ND	ND	ND	ND	ND	ND	ND	ND	ND	ND	ND	ND	ND	ND
2,3,4,7,8-PeCDF	0.02	0.02	0.02	0.02	0.01	0.02	0.01	0.02	0.01	0.02	0.01	0.02	ND	0.02	0.01	0.02	ND	0.02
1,2,3,7,8-PeCDD*	0.07	0.10	0.05	0.06	0.02	0.09	0.08	0.06	0.11	0.05	0.08	0.05	0.10	0.05	0.11	0.06	0.12	0.05
1,2,3,4,7,8-HxCDF*	ND	ND	0.01	0.01	ND	0.01	ND	0.01	ND	0.01	ND	0.01	ND	0.01	ND	0.01	ND	0.01
1,2,3,6,7,8-HxCDF	ND	ND	0.01	0.01	ND	0.01	ND	0.01	ND	0.01	ND	0.01	ND	0.01	ND	0.01	ND	0.01
2,3,4,6,7,8-HxCDF*	ND	ND	0.01	0.01	ND	0.01	ND	0.01	ND	0.01	ND	0.01	ND	0.01	ND	0.01	ND	0.01
1,2,3,7,8,9-HxCDF	ND	ND	0.01	0.01	ND	0.01	ND	0.01	ND	0.01	ND	0.01	ND	0.01	ND	0.01	ND	0.01
1,2,3,4,7,8-HxCDD*	0.01	ND	0.01	0.01	ND	0.01	ND	0.01	0.01	0.01	0.01	0.01	ND	0.01	ND	0.01	0.01	0.01
1,2,3,6,7,8-HxCDD	0.01	ND	0.01	0.01	ND	0.01	ND	0.01	0.01	0.01	0.01	0.01	ND	0.01	ND	0.01	ND	0.01
1,2,3,7,8,9-HxCDD	ND	0.01	0.01	0.01	ND	0.01	ND	0.01	ND	0.01	ND	0.01	ND	0.01	ND	0.01	ND	0.01
1,2,3,4,6,7,8-HpCDF*	ND	ND	ND	ND	ND	ND	ND	ND	ND	ND	ND	ND	ND	ND	ND	ND	ND	ND
1,2,3,4,7,8,9-HpCDF	ND	ND	ND	ND	ND	ND	ND	ND	ND	ND	ND	ND	ND	ND	ND	ND	ND	ND
1,2,3,4,6,7,8-HpCDD*	ND	ND	ND	ND	ND	ND	ND	ND	ND	ND	ND	ND	ND	0.01	ND	ND	ND	ND
OCDF*	ND	ND	ND	ND	ND	ND	ND	ND	ND	ND	ND	ND	ND	ND	ND	ND	ND	ND
OCDD*	ND	ND	ND	ND	ND	ND	ND	ND	ND	ND	ND	ND	ND	ND	ND	ND	ND	ND
Total	0.13	0.16	0.16	0.17	0.04	0.20	0.12	0.17	0.16	0.16	0.12	0.16	0.11	0.17	0.17	0.78	0.14	0.16

*All data are expressed as means of two replicates in pg WHO-TEQ/g fresh weight. The TEFs of TCDF, TCDD, PeCDF, PeCDD, HxCDF, HxCDD, HpCDF, HpCDD, OCDF and OCDD are 0.1, 1.0, 0.03/0.3, 1.0, 0.1, 0.1, 0.01, 0.01, 0.0003 and 0.0003, respectively. SP: Silver pomfret; HS: hardtail scad; FT: fourfinger threadfin; IM: Indian mackerel; LTS: large-scale tongue sole; LBR: long-tailed butterfly ray; Cf: cuttlefish; JTB: Japanese threadfin bream; SG: sixbar grouper; TCDF: tetrachlorodibenzofuran; TCDD: tetrachlorodibenzodioxin; PeCDF: pentachlorodibenzofuran; PeCDD: pentachlorodibenzodioxin; HxCDF: hexachlorodibenzofuran; HxCDD: hexachlorodibenzodioxin; HpCDF: heptachlorodibenzofuran; HpCDD: heptachlorodibenzodioxin; OCDF: octachlorodibenzofuran; OCDD: octachlorodibenzodioxin; ND: not detected

**Table 2:** Congeners of PCDD/PCDF in fish and shellfish samples from trip 1 (T1) and trip 2 (T2) of middle region

Congeners	MRS*		HS*		FTB*		IM*		LBR*		Pr*		SG*		Cf*		Ck*	
	T1	T2	T1	T2	T1	T2	T1	T2	T1	T2	T1	T2	T1	T2	T1	T2	T1	T2
2,3,7,8-TCDF	0.01	0.02	ND	0.02	ND	ND	ND	0.02	ND	ND	ND	ND	ND	0.01	ND	ND	0.01	ND
2,3,7,8-TCDD	0.05	0.07	0.03	0.05	0.02	0.02	0.02	0.05	0.02	0.02	0.02	0.02	0.02	0.06	0.02	0.03	0.07	0.04
1,2,3,7,8-PeCDF	ND*	ND	ND	ND	ND	ND	ND	ND	ND	ND	ND	ND	ND	ND	ND	ND	ND	ND
2,3,4,7,8-PeCDF	0.03	0.05	0.04	0.03	0.02	0.02	0.02	0.03	0.02	0.02	0.02	0.02	0.02	0.02	0.02	0.02	0.02	0.02
1,2,3,7,8-PeCDD	0.22	0.20	0.13	0.09	0.06	0.12	0.07	0.09	0.05	0.05	0.09	0.13	0.05	0.12	0.05	0.05	0.09	0.10
1,2,3,4,7,8-HxCDF	0.01	0.01	0.01	0.01	0.01	0.01	0.01	0.01	0.01	0.01	0.01	0.01	0.01	0.01	0.01	0.01	0.01	0.01
1,2,3,6,7,8-HxCDF	0.01	0.01	0.01	0.01	0.01	0.01	0.01	0.01	0.01	0.01	0.01	0.01	0.01	0.01	0.01	0.01	0.01	0.01
2,3,4,6,7,8-HxCDF	0.01	0.01	0.01	0.01	0.01	0.01	0.01	0.01	0.01	0.01	0.01	0.01	0.01	0.01	0.01	0.01	0.01	0.01
1,2,3,7,8,9-HxCDF	0.01	0.01	0.01	0.01	0.01	0.01	0.01	0.01	0.01	0.01	0.01	0.01	0.01	0.01	0.01	0.01	0.01	0.01
1,2,3,4,7,8-HxCDD	0.01	0.01	0.01	0.01	0.01	0.01	0.01	0.01	0.01	0.01	0.01	0.01	0.01	0.01	0.01	0.01	0.01	0.01
1,2,3,6,7,8-HxCDD	0.01	0.01	0.01	0.01	0.01	0.01	0.01	0.01	0.01	0.01	0.01	0.01	0.01	0.01	0.01	0.01	0.01	0.01
1,2,3,7,8,9-HxCDD	0.01	0.01	0.01	0.01	0.01	0.01	0.01	0.01	0.01	0.01	0.01	0.01	0.01	0.01	0.01	0.01	0.01	0.01
1,2,3,4,6,7,8-HpCDF	ND	ND	ND	ND	ND	ND	ND	ND	ND	ND	ND	ND	ND	ND	ND	ND	ND	ND
1,2,3,4,7,8,9-HpCDF	ND	ND	ND	ND	ND	ND	ND	ND	ND	ND	ND	ND	ND	ND	ND	ND	ND	ND
1,2,3,4,6,7,8-HpCDD	ND	ND	ND	ND	ND	ND	ND	ND	ND	ND	ND	ND	ND	0.01	ND	ND	0.01	ND
OCDF	ND	ND	ND	ND	ND	ND	ND	ND	ND	ND	ND	ND	ND	ND	ND	ND	ND	ND
OCDD	ND	ND	ND	ND	ND	ND	ND	ND	ND	ND	ND	ND	ND	ND	ND	ND	ND	ND
Total	0.38	0.41	0.27	0.26	0.17	0.23	0.18	0.26	0.16	0.16	0.20	0.24	0.16	0.29	0.16	0.17	0.27	0.23

*All data are expressed as means of two replicates in pg WHO-TEQ/g fresh weight. MRS: Malabar red snapper; HS: hardtail scad; FTB: fourfinger threadfin; IM: Indian mackerel; LBR: long-tailed butterfly ray; Pr: prawn; SG: sixbar grouper; Cf: cuttlefish; Ck: cockles; ND: not detected

**Table 3:** Congeners of PCDD/PCDF in fish and shellfish samples from trip 1 (T1) and trip 2 (T2) of southern region

Congeners	DW*		HS*		SM*		GEC*		Cf*		Pr*	
	T1	T2	T1	T2	T1	T2	T1	T2	T1	T2	T1	T2
2,3,7,8-TCDF	0.01	0.01	0.01	ND	0.02	ND	ND	0.01	ND	ND	ND	ND
2,3,7,8-TCDD	0.04	0.02	0.04	0.02	0.04	0.02	0.06	0.11	0.02	0.02	0.04	0.04
1,2,3,7,8-PeCDF	ND*	ND	ND	ND	ND	ND	ND	ND	ND	ND	ND	ND
2,3,4,7,8-PeCDF	0.02	0.02	0.04	0.02	0.05	0.02	0.03	0.07	0.02	0.02	0.02	0.02
1,2,3,7,8-PeCDD	0.07	0.05	0.27	0.10	0.22	0.13	0.54	1.04	0.05	0.07	0.15	0.19
1,2,3,4,7,8-HxCDF	0.01	0.01	0.01	0.01	0.01	0.01	0.01	0.01	0.01	0.01	0.01	0.01
1,2,3,6,7,8-HxCDF	0.01	0.01	0.01	0.01	0.01	0.01	0.01	0.01	0.01	0.01	0.01	0.01
2,3,4,6,7,8-HxCDF	0.01	0.01	0.01	0.01	0.01	0.01	0.01	0.01	0.01	0.01	0.01	0.01
1,2,3,7,8,9-HxCDF	0.01	0.01	0.01	0.01	0.01	0.01	0.01	0.01	0.01	0.01	0.01	0.01
1,2,3,4,7,8-HxCDD	0.01	0.01	0.01	0.01	0.01	0.01	0.05	0.06	0.01	0.01	0.01	0.01
1,2,3,6,7,8-HxCDD	0.01	0.01	0.01	0.01	0.01	0.01	0.09	0.11	0.01	0.01	0.01	0.01
1,2,3,7,8,9-HxCDD	0.01	0.01	0.01	0.01	0.01	0.01	0.08	0.10	0.01	0.01	0.01	0.01
1,2,3,4,6,7,8-HpCDF	ND	ND	ND	ND	ND	ND	ND	ND	ND	ND	ND	ND
1,2,3,4,7,8,9-HpCDF	ND	ND	ND	ND	ND	ND	ND	ND	ND	ND	ND	ND
1,2,3,4,6,7,8-HpCDD	ND	ND	ND	ND	ND	ND	0.02	0.03	ND	ND	ND	ND
OCDF	ND	ND	ND	ND	ND	ND	ND	ND	ND	ND	ND	ND
OCDD	ND	ND	ND	ND	ND	ND	ND	ND	ND	ND	ND	ND
Total	0.21	0.17	0.43	0.21	0.40	0.24	0.91	1.57	0.16	0.18	0.28	0.32

*All data are expressed as means of two replicates in pg WHO-TEQ/g fresh weight. DW: dorab wolf-herring; HS: hardtail scad; SM: Spanish mackerel; GEC: grey eel-catfish; Cf: cuttlefish; Pr: prawn; ND: not detected

Among the 17 congeners of PCDD/PCDF determined, 1,2,3,7,8-PeCDD was the most abundant congener found in all samples, which ranged between 0.02 and 1.04 pg WHO-TEQ/g FW compared with the other congeners. The results were in agreement with the data reported previously [22], where 1,2,3,7,8-PeCDD contributed to 21-78 % of the WHO-TEQ. Similar findings were also reported previously, where the 1,2,3,7,8-PeCDD congener contributed to about 31 % of the WHO-TEQ [23]. According to WHO, 1,2,3,7,8-PeCDD has TEF value of 1.0 [18]. Additionally, 2,3,7,8-TCDD has TEF value of 1.0. It was classified as the Group 1 carcinogen (human carcinogen) by the WHO’s International Agency for Research on Cancer in 1997.

The congeners, 2,3,7,8-TCDD detected in all studied samples ranged from 0.01 to 0.11 pg WHO TEQ/g FW. The mean concentration of 2,3,7,8-TCDD congener (0.02 pg WHO-TEQ/g FW) in the Malaysian seafood samples determined previously was within the range of the concentration found in this study. In addition, the congeners including 1,2,3,4,6,7,8-HpCDF, 1,2,3,4,7,8,9-HpCDF, OCDF and OCDD were not detected in any of the samples. One of the reasons is these congeners are poorly absorbed by the digestive tract of fish and shellfish, where the degree of chlorination in these congeners are higher compared with that of the other congeners [23, 24].

The types of fish that contained the highest WHO-TEQ levels were Japanese threadfin bream, large-scale tongue sole, fourfinger threadfin, Malabar red snapper, cockles, sixbar grouper and grey eel-catfish. The results showed that the highest concentrations of 2,3,7,8-TCDD, 1,2,3,7,8-PeCDD and 1,2,3,4,7,8-HxCDD were detected in grey eel-catfish samples obtained from the southern region (Table 3). Among all samples, Malabar red snapper had the highest concentration of 2,3,4,7,8-PeCDF congener (0.05 pg WHO_TEQ/g FW) from the middle region during T1’s sample collection. Based on the results obtained, the congener profiles are species-dependent. The results could have also been influenced by biological (metabolism, age and trophic level) and environmental factors (habitat, geography and seasonal variation) [23, 25].

Congener 2,3,4,7,8-PeCDF has shown to be responsible for about 70 % of the dioxin toxicity, and it has been identified as an important causative agent in Yusho disease [26]. The 2,3,4,7,8-PeCDF is also reported to be the second most potent and toxic congener after 2,3,7,8-TCDD [20]. Similar pattern of the congener profile of the seafood sample was reported previously [27], in which the largest contribution to the PCDD/PCDF toxicity was from 2,3,4,7,8-PeCDF, 2,3,7,8-TCDF, 1,2,3,7,8-PeCDD and 2,3,7,8-TCDD.

In this study, four main congeners were detected in all seafood samples. The levels of all congeners in the seafood samples were below 1 pg WHO-TEQ/g FW with some exception. The presence of specific PCDD/PCDF congeners in certain seafood samples could be related to industrial activities nearby the sea where the fish and shellfish samples were collected. Since the fish and shellfish samples collected along the Strait of Malacca have indicated some contamination with PCDD/PCDF congeners, the relevant authority in Malaysia recommended to monitor disposal of waste from factories nearby the Straits.

The concentrations of PCDD/PCDF (WHO-TEQ) in the fish and shellfish samples obtained from different regions along the Straits are presented in Table 4. The results showed that grey eel-catfish (southern region), Japanese threadfin bream (northern region) and Malabar red snapper (middle region) contained the highest PCDD/PCDF concentrations, with concentrations of 1.24, 0.46 and 0.36 pg WHO-TEQ/g FW, respectively. On the other hand, fourfinger threadfin bream from the northern region had the lowest PCDD/PCDF concentrations at 0.12 pg/g FW, respectively. A high WHO-TEQ was determined for grey eel-catfish because it is the most affected species that exhibits the highest total PCDD/PCDF at 1.24 ± 0.47 pg WHO-TEQ/g FW compared with other samples. Among the fish and shellfish samples of different regions, no significant differences were found for the WHO-TEQ levels of all types of the samples, which could be due to the fact that sea creatures freely move along the Straits of Malacca.

**Table 4:** Total PCDD/PCDF in fish and shellfish along the Straits of Malacca by regions

Sample	WHO-TEQ (pg/g fresh weight)			Mean fat content (%)	p-value*
	Northern region	Middle region	Southern region		
Hardtail scad	0.17 ± 0.01	0.27 ± 0.01	0.32 ± 0.17	1.9	0.360
Spanish mackerel	-	-	0.32 ± 0.15	4.4	-
Grey eel-catfish	-	-	1.24 ± 0.47	5.7	-
Dorab wolf-herring	-	-	0.19 ± 0.04	3.7	-
Fourfinger threadfin	0.12 ± 0.08	0.2 ± 0.04	-	2.5	0.473
Indian mackerel	0.15 ± 0.01	0.22 ± 0.07	-	3.5	0.316
Japanese threadfin bream	0.48 ± 0.41	-	-	2.3	-
Long-tailed butterfly ray	0.14 ± 0.02	0.16 ± 0.01	-	1.1	0.333
Sixbar grouper	0.15 ± 0.01	0.23 ± 0.09	-	2.9	0.429
Silver pomfret	0.15 ± 0.02	-	-	3.7	-
Large-scale tongue sole	0.16 ± 0.03	-	-	0.8	-
Malabar red snapper	-	0.40± 0.02	-	5.5	-
Cockles	-	0.25 ± 0.02	-	3.0	-
Prawn	-	0.22 ± 0.03	0.30 ± 0.04	1.3	0.117
Cuttlefish	0.14 ± 0.01	0.17 ± 0.01	0.17 ± 0.02	2.6	0.604

*The total PCDD/PCDF (mean of total congeners) of the samples were not significantly different (p ≥ 0.05) among the regions

Our previous study [28] demonstrated that the levels of total dioxin and furan in these species of fish and shellfish from the Straits of Malacca were high. The results also showed that grey eel-catfish obtained from the southern region of the Straits of Malacca during trip 2 had the highest level of total PCDD/PCDF (1.57 pg WHO-TEQ/g FW) compared with other seafood samples. In this study, the WHO-TEQ level (1.24 pg/g FW) for grey eel-catfish was similar to the result reported previously. One possible explanation for the high WHO-TEQ level could be that grey eel-catfish ingested these toxic substances during food intake from the muddy ocean floor. On the other hand, the total PCDDs/PCDFs (pg WHO-TEQ/g FW) for fish fillet samples of Indian mackerel (0.10), silver pomfret (0.13), grey eel-catfish (1.23), hardtail scad (0.12) and Spanish mackerel (0.18) as reported by Azrina *et al.* [29] are lower than the WHO-TEQ levels determined in this study (Table 4). Conversely, we found the total PCDD/PCDF in the fish and shellfish samples ranged between 4.6 and 21.8 pg WHO-TEQ/g fat. Therefore, it is important to monitor the levels of PCDD/PCDF in the seafood samples obtained from the Straits of Malacca on a regular basis.

A recent study in Malaysia reported that the mean levels of PCDD/PCDF in eight types of seafood (tilapia, grouper, pomfret, barramundi, horse mackerel, snapper, prawn and cuttlefish) ranged from 0.16 to 0.17 pg WHO-TEQ/g FW [23]. The results were much lower than the concentrations of PCDD/PCDF of the same species determined in this study. It could be due to the homogenous edible portions of the sample analysed as a group of seafood that were not determined according to individual species. As reported in another study, the total PCDD/PCDF in fish and shellfish samples from Catalan market, Spain, ranged from 0.11 to 0.66 pg WHO-TEQ/g FW [30]. The results obtained from this study showed that the seafood samples obtained from the West Coast of Peninsular Malaysia along the Straits contained higher concentrations of PCDD/PCDF than the samples from the coastal areas of Japan. However, Moon and Ok [31] reported that the concentrations of PCDD/PCDF in 40 types of seafood samples from Korean coastal area ranged from 0.02 to 4.39 pg WHO-TEQ/g FW. The TEQ values recorded by Moon and Ok [31] were higher than the TEQ values found in our study. Based on the wet weight of the samples, all these findings revealed the total PCDD/PCDF detected.

On the other hand, the concentrations of PCDD/PCDF in aquatic food obtained from the local market in China ranged from 0.9 to 15317 pg WHO-TEQ/g fat [32]. Based on a previous study, meat and poultry from Belgium have TEQ levels (PCDDs/PCDFs) ranging from trace to 7.82 pg WHO-TEQ/g fat [33]. The results showed that horse meat has the highest total PCDD/PCDF (7.82 pg WHO-TEQ/g fat), followed by eggs (2.76 pg/g), beef and mutton (1.56 and 1.55 pg/g). Also, pork and chicken meat contained the lowest WHO-TEQ levels, 0.17 and 0.35 pg/g fat, respectively. This finding demonstrates that pork and chicken meat contain lower total PCDD/PCDF than the fish and shellfish samples.

The safe level of PCDD/PCDF in food is about 1 pg WHO-TEQ/g fat [17]. Therefore, seafood samples from the Straits of Malacca are not safe for consumption as the total PCDD/PCDF was higher than 1 pg WHO-TEQ/g fat. In addition to food products, a high total PCDD/PCDF/PCB (13 pg WHO-TEQ/g fat) was also found in the breast milk of mothers who lived in the northern region of Peninsular Malaysia nearby the Strait of Malacca [34]. The TEQs in the breast milk of mothers ranged from 3.0 to 24.0 pg WHO-TEQ/g fat. One possible reason for the high TEQ of mother breast milk is these mothers have consumed contaminated meat and seafood.

The rapid growth of agricultural and industrial sectors, as well as urbanisation on the west coast of Peninsular Malaysia, are among the contributors of these POP [35]. The northern, middle and southern regions of the west coast have different stages of development and industrialisation, which might have contributed to the release of PCDD/PCDF to the environment [36]. The smoke emitted from the human activities contains PCDD or PCDF, which increase the levels of PCDD/PCDF in the seawater.

### Correlation between fat contents and levels of PCDD/PCDF

Fat contents of the 48 samples of fish and shellfish ranged between 0.80 % and 5.70 %. The result of statistical analysis showed a positive moderate correlation between fat content and concentrations of PCDD/PCDF in the seafood samples (r = 0.507; p < 0.05). The moderately high correlation shows that about half of the PCDD/PCDF exposure could be due to the intake of fat from the fish and shellfish. Although dioxin and furan are lipophilic and highly soluble in fat, the moderate correlation is owing to the reason that some of the shellfish samples contained a low amount of fat.

### Sociodemographic, anthropometric and lifestyle characteristics of respondents

The sociodemographic characteristics, anthropometric and blood pressure measurements, as well as the lifestyle of the 93 recruited fishermen (respondents), are shown in Table 5. A majority of the respondents were male (81.7 %). The age of more than 50 % of the respondents was 40 or higher. They came from Malay, Chinese and Indian ethnic groups and a majority of them were Malay (57 %). Most of the respondents in the fisherman community had monthly income below the poverty line, where the monthly incomes of more than 90 % of the respondents were below RM 1,500 (equivalent to about £ 270). In Selangor State, Malaysia, RM 1,500 has been set as the poverty line [37]. Some of the fishermen are illiterate, while most of them had completed primary school. A considerable proportion (91 %) of these fishermen were experienced with over 15 years of working experience. On average, the BW and height of the respondents were 67.38 kg and 162.95 cm, respectively. They also had ideal BW on average, with body fat percentages and BMI of 26.01 % and 25.24 kg/m^2^, respectively. The systolic and diastolic blood pressures of the respondents were 138.82 and 80.53 mm Hg, respectively. In addition, the majority of the respondents did not smoke cigarettes or drink alcoholic beverages. With regard to cigarette smoking, it is banned by most of the religions.

**Table 5:** Sociodemographic characteristics, anthropometric measurements and lifestyle of fishermen

Characteristics	n = 93 (%)	Mean ± SD	Range
Gender			
Male	76 (81.7)		
Female	17 (18.3)		
Age group (years old)		46.52 ± 13.55	13-81
<19	3 (3.2)		
20-29	5 (5.4)		
30-39	17 (18.3)		
40-49	32 (34.4)		
>50	36 (38.7)		
Race			
Malay	53 (57.0)		
Chinese	39 (41.9)		
Indian	1 (1.1)		
Household income (RM)		1144.73 ± 770.39	250-4000
<500	18 (19.4)		
500-1000	26 (27.9)		
1001-1500	42 (45.2)		
>1500	7 (7.5)		
Education level			
Formal education	88 (94.6)		
No formal education	5 (5.4)		
Working experience (years)		–	–
10–15	9 (12.9)		
>15	84 (91.1)		
Working hours (h/week)		39.8 ± 16.0	6-120
Anthropometric			
Weight (kg)		67.38 ± 14.08	41.5-118.1
Height (cm)		162.95 ± 8.65	131.0-182.0
Fat percentage (%)		26.01 ± 8.29	11.8-56.2
BMI (kg/m^2^)		25.24 ± 4.75	16.7-40.9
Blood pressure (mm Hg)			
Systolic		138.82 ± 21.77	94-207
Diastolic		80.53 ± 13.19	45-115
Smoking		–	1-20 cigarette/day
Smoker	35 (37.6)		
Non-smoker	52 (55.9)		
Already quit	6 (6.5)		
Alcohol consumption		–	–
Yes	13 (14.0)		
No	78 (83.9)		
Already quit	2 (2.1)		

* SD: standard deviation

### Dietary intake and PCDD/PCDF exposure

The percentages of fish and shellfish consumption among the respondents are presented in Table 6. The seafood consumption data were collected based on daily, weekly and monthly basis using a food frequency questionnaire. The information was limited to only the frequency of consumption of specific seafood caught along the Straits of Malacca where the sample was obtained. As shown in Table 6, 15 types of fish and shellfish were considered for the seafood intake section in the questionnaire. The percentages of fish and shellfish intake were calculated based on a daily basis. The results showed that prawn (11.8 %) was the most frequently consumed seafood among the respondents, followed by fourfinger threadfin (6.5 %) and Indian mackerel (6.5 %). Indian mackerel and prawn were also found to be highly consumed by the respondents on a weekly basis, accounting for about 59.1 % and 52.7 % of intake, respectively. Meanwhile, on a monthly basis, long-tail butterfly ray (44.1 %), cuttlefish (44.1 %) and hardtail scad (40.9 %) were the major seafood consumed.

**Table 6:** The percentage fish and shellfish consumed by respondents

Sample	Daily (%)	Weekly (%)	Monthly (%)
Hardtail scad	-	26.9	40.9
Spanish mackerel	1.1	18.3	36.6
Grey eel-catfish	-	30.1	27.9
Dorab wolf-herring	2.2	11.8	13.9
Fourfinger threadfin	6.5	36.6	25.8
Indian mackerel	6.5	59.1	18.3
Japanese threadfin bream	1.1	6.5	13.9
Long-tailed butterfly ray	-	37.6	44.1
Sixbar grouper	2.2	10.8	16.1
Silver pomfret	-	30.1	35.5
Large-scale tongue sole	4.3	39.8	31.2
Malabar red snapper	1.1	16.1	22.6
Cockles	4.3	21.5	38.7
Prawn	11.8	52.7	29.0
Cuttlefish	4.3	21.5	44.1

Average intakes of a specific type of fish or shellfish, as well as total fish intake by the respondents, are presented in Table 7. The amount of large-scale tongue sole fillet consumed by the respondents was the highest among all seafood samples, with a value of 44.06 ± 97.10 g/person/day. The amounts of fish and shellfish consumed by the respondents ranged from 2.02 ± 0.87 to 44.06 ± 10.07 g/person/day. The amounts of seafood consumption reported in this study are much lower than the amounts reported in the Food Consumption Statistics of Malaysia 2003 [38], of which the estimated mean intake of seafood for Malaysian population is 60.67 g/day and 75.59 g/day for rural areas.

**Table 7:** Average seafood consumption (ASC) of fish/shellfish and PCDD/PCDF exposure among fishermen and family members

Sample	ASC (g/person/day)*	PCDD/PCDF exposure (pg WHO-TEQ/kg BW/day)*
Hardtail scad	19.13 ± 3.89	0.12 ± 0.03
Spanish mackerel	13.02 ± 2.98	0.07 ± 0.02
Grey eel-catfish	6.71 ± 1.12	0.16 ± 0.03
Dorab wolf-herring	10.14 ± 3.30	0.03 ± 0.01
Fourfinger threadfin	17.31 ± 4.72	0.05 ± 0.01
Indian mackerel	20.87 ± 3.15	0.07 ± 0.01
Japanese threadfin bream	2.02 ± 0.87	0.02 ± 0.01
Long-tailed butterfly ray	22.64 ± 3.09	0.06 ± 0.01
Sixbar grouper	4.46 ± 1.46	0.02 ± 0.01
Silver pomfret	8.95 ± 1.46	0.02 ± 0.00
Large-scale tongue sole	44.06 ± 10.07	0.13 ± 0.30
Malabar red snapper	7.60 ± 2.05	0.04 ± 0.01
Cockles	2.64 ± 0.58	0.01 ± 0.00
Prawn	12.14 ± 2.09	0.05 ± 0.01
Cuttlefish	11.78 ± 3.80	0.03 ± 0.01

*All data are presented as mean ± standard error of the mean

Previously, a high intake of fish and seafood products among Malaysian has been reported at 103.7 g/day [23]. The study has also included other fishery products. However, it focuses on the consumption of 15 types of fish and shellfish among the respondents. The intake of seafood by respondents depended on the availability of these fish and shellfish; therefore, not all types of the seafood were considered. The estimated value of marine fish sources for the general population stated in the Food Consumption Statistics of Malaysia, 2003 [38] does not indicate the consumption of particular types of fish or shellfish. Therefore, the result obtained can be used as a guideline for consumption of selected fish and shellfish among the fishing community in the middle region of the east coast of Peninsular Malaysia.

Table 7 shows the average PCDD/PCDF exposure (pg WHO-TEQ/kg BW/day) of the respondents. The PCDD/PCDF concentration of each sample was used to calculate the average PCDD/PCDF exposure (pg WHO-TEQ/kg BW/day). The highest average exposure to total PCDD/PCDF (pg WHO-TEQ/kg BW/day) among the respondents was attributed to grey eel-catfish (0.16), followed by hardtail scad (0.12) and large-scale tongue sole (0.13). Total PCDD/PCDF exposures from consumption of fish and shellfish among the respondents ranged from 0.01 to 0.16 pg WHO-TEQ/kg BW/day. In Malaysia, seafood and seafood products have contributed to PCDD/PCDF exposure at 0.41 pg WHO-TEQ/kg BW/day [23], which is higher than the exposure among the respondents in this study. The high level of PCDD/PCDF exposure is mainly due to the consumption of fish, shellfish and also other seafood products such as canned sardine, canned crab meat, fish ball, tempura seafood, crab stick and others that are contaminated with PCDD/PCDF. In this study, the dietary exposure to PCDD/PCDF from seafood intake among the respondents was low. It is because the ASC only covered daily intake of selected fish and shellfish species among the fishermen. The PCDD/PCDF exposure among the respondents was much lower than the exposure reported by studies from Egypt (4.06-6.38 pg TEQ/kg BW/day) [39], China (1.36 pg TEQ/kg BW/day) [40] and Spain (1.17 pg TEQ/kg BW/day) [41].

The results obtained from this study could represent the safety level of PCDD/PCDF in the 15 types of fish and shellfish from the Straits of Malacca. Previous research revealed that the levels of PCDD/PCDF in the serum lipid profile of fishermen were within the range of 70-200 pg WHO-TEQ/g lipid, with high consumption of Baltic fish and shellfish [42]. The study also reported that the fishermen who consumed low to moderate amounts of Baltic seafood have 30-140 pg WHO-TEQ/g lipid detected in the serum. Therefore, increased consumption of seafood has contributed to a high exposure of PCDD/PCDF.

Among the 93 respondents, 23 of them detected having skin disease by a dermatologist. The types of skin disease detected are reported in Table 8. Besides the exposure to PCDD/PCDF, some of the skin diseases could be caused by other factors, such as sun exposure [43] and microbial infection [44]. The occurrence of skin diseases was high among fishermen because they worked in the environmental conditions that promote exposure to contaminants [45]. Except for hyperpigmentation, the other types of skin disease were not related to PCDD/PCDF poisoning. There was also no chloracne case detected in this study. Skin diseases occur due to high and mostly accidental intakes of PCDD/PCDF. Therefore, skin disorders could not be expected among the respondents.

**Table 8:** Type of skin disease among fishermen and family members

Skin disease	No. of respondent (n = 93)	Percentage (%)
Occurrence		
No	70	75.0
Yes	23	25.0
Types		
Eczema	7	7.6
Tinea versicolor (Tinea)	4	4.3
Psoriasis	3	3.2
Hyperpigmentation	2	2.2
Tinea cruris/ corporis (Ringworm)	2	2.2
Acne vulgaris	1	1.1
Seborrheic capitis (Dandruff)	1	1.1
Tinea corporis (Ringworm)	1	1.1
Toe-web intertrigo	1	1.1
Sebaceous cyst	1	1.1
Seborrhoeic wart	0	0
Favre-Racouchot syndrome	0	0
Hyperpigmentation		
Skin (trunk)	1	1.1
Mucosa (cheek and gum)	1	1.1

Chloracne and hyperpigmentation are the two most common types of skin disease related to PCDD/PCDF exposure to humans. The study merely focused on skin diseases related to PCDD/PCDF exposure among the respondents. As shown in Table 8, two respondents (2.2 %) were found to have hyperpigmentation. Only one of them had skin hyperpigmentation, whereas the other had hyperpigmentation on the mucosa (gum and buccal mucosa). It is highly unlikely that this localised hyperpigmentation is caused by dioxins/furans exposure or toxicity. All cases of dioxin/furan-related hyperpigmentation reported a severe, generalised darkening of skin and abnormal pigmentation, with almost the entire body surface area involved [25]. Ideally blood levels of dioxins/furans or congeners in these two fishermen should be measured, which would confirm the relationship of the skin changes and dioxins/furans toxicity. However, the hyperpigmentation detected among the respondents could be due to extreme exposure to sunlight while fishing [43]. Based on their fish intake assessed using FFQ, both of the respondents had low PCDD/PCDF exposure. The first and second respondents who were diagnosed with hyperpigmentation consumed 8.63 and 13.61 g of fish per day, respectively. The exposures to PCDD/PCDF for both of them were 0.04 and 0.06 pg WHO-TEQ/kg BW/day, respectively. The low level of exposure reaffirms the finding that the skin hyperpigmentation is unrelated to PCDD/PCDF. Also, the exposure was below the recommended level (1 pg TEQ/kg BW/day) [16].

Sociodemographic characteristics and lifestyles could be the main factors for hyperpigmentation of the skin. The low exposure to PCDD/PCDF for the two respondents shows that dioxin and furan toxicity was not the cause of skin hyperpigmentation. Sunlight exposure seems to be the only cause of hyperpigmentation. On the contrary, the members of a Spanish family (father, mother and six children) developed chloracne and hyperpigmentation due to ingestion of olive oil contaminated with PCDD/PCDF [46]. The level of PCDD/PCDF detected in the chloracneigenic oil from Spain was 1590 pg WHO-TEQ/g oil, where the levels of exposure to these contaminants among the family member ranged from 620 to 1500 pg WHO-TEQ/kg BW/day. Hyperpigmentation and acne-like eruptions have also been documented in the “Yusho” incidence in Japan. The incidence was caused by ingestion of Japanese rice oil containing PCB and PCDF at the exposure level of <400 ppm [25].

The results obtained from this study revealed that the low PCDD/PCDF exposure among the respondents indicates the skin diseases cannot be contributed by PCDD/PCDF toxicity. Based on the previous literature, a high level of exposure is required to cause skin toxicity (chloracne and hyperpigmentation) [47]. Based on a follow-up study reported by Guo *et al.* [48], the subjects who had emitted to hospital were estimated to have consumed about 3.8 mg of PCDFs. The amounts of PCDD/PCDF ingested by the respondents were estimated to be more than 2000 times lower than the reported case.

Exposure to PCDD/PCDF through intake of fish and shellfish is considered one of the risk factors for skin toxicity. Although blood samples were not taken from the respondents, the information obtained based on the questionnaire, as well as the skin examination by the dermatologist, could provide some hints on the possible contribution of dietary fish and shellfish to skin diseases related to PCDD/PCDF toxicity. The information is very important in the future as guidelines to the authority and public, as well as research scientists, for further investigation of PCDD/PCDF exposure among the fishing community since this group is more likely to be affected by environmental pollutants. Additionally, this study will provide baseline data to the stakeholders in Malaysian fishery industry. The data can also be used as a guideline to determine whether the levels of PCDD/PCDF in local marine fish and shellfish are below the recommended levels [17].

## Conclusion

Contamination of fish and shellfish with PCDD and PCDF is hazardous to those who consumed the contaminated seafood. PCDD/PCDF exposure among the fishermen recruited from the selected fishing villages in the coastal area of the Straits of Malacca is low. Almost all the fish and shellfish samples had less than 0.5 pg WHO-TEQ/g fresh weight, indicating that the seafood obtained from the Strait of Malacca has low PCDD/PCDF levels. However, the total PCDD/PCDF levels in all the fish and shellfish samples were higher than 1.0 WHO-TEQ/g fat. It is somehow not safe for consumption. Although the Straits is one of the busiest sea routes for international traders, the level of seawater pollution level is still low. Due to the high levels of PCDD/PCDF congeners in the seafood obtained from the southern region during the second trip, continuous monitoring of human activities is essential. Authorities from the nearby countries should monitor the related human activities that could have polluted the seawater of the Straits. Monitoring the levels of dioxin and furan from time to time should be carried out to ensure safe seafood products for the community.
